# A mixed methods study on the association between burnout symptoms and expectations regarding clerkships in Dutch medical students

**DOI:** 10.12688/mep.20633.1

**Published:** 2025-07-18

**Authors:** Abraham M. Rubens, Rozemarijn van der Gulden, Marjolein H.J. van de Pol

**Affiliations:** 1 Primary and Community Care, Radboud University Medical Center, Nijmegen, 6500HB, The Netherlands

**Keywords:** Burnout, Students, Expectation mismatch, clerkships, medicine

## Abstract

**Background:**

Over half of the Dutch students experience burnout symptoms. In order to design effective interventions, it is important to know more about cognitions and behaviors that relate to burnout. An example of such cognitions might be inaccurate expectations of medical students regarding their study, as studies in other populations already indicated that unmet expectations can relate to burnout symptoms. Therefore, the aim of this study is to evaluate the relationship between an expectation-reality mismatch of medical students and burnout symptoms.

**Methods:**

We performed an exploratory mixed methods study consisting of two sequential data collection methods. Firstly, individual semi-structured interviews were performed with six students to explore expectations. Secondly, we designed an anonymous questionnaire to explore presence of expectation mismatches and the relationship with burnout symptoms. Burnout was assessed using the short Burnout Assessment Tool (sBAT).

**Results:**

155 medical students filled in the questionnaire. Important mismatch topics were: level of supervision, feedback quality, amount of time spent on different tasks, amount of initiative needed and culture on clerkship location. We found that 21.9% of the students scored high to very high on the sBAT. Moreover, a mismatch between expectations and reality was positively correlated with burnout.

**Conclusions:**

Students that experienced a bigger mismatch between expectations and reality also experienced more burnout symptoms. Consequently, it is important that future research further establishes this relationship, as expectation management might be an interesting avenue to reduce negative experiences and emotions during the clerkships.

## Background

Recent research shows that over half of the students in the Netherlands experience burnout symptoms and a high level of stress ^1^. These numbers are in line with those of medical students from other countries ^2, 3^. These alarming numbers cause serious concerns about the wellbeing of medical students ^4, 5^. Consequently, universities are trying to find new interventions to prevent burnout and stress related symptoms. However, in order to design effective interventions it is important to know which cognitions and behaviors relate to burnout. While previous research already showed that perfectionism, maladaptive coping behavior and lack of physical exercise are related to burnout of medical students ^6, 7^, other factors that might relate to burnout are underexplored.

The inaccurate expectations of medical students regarding their study might be such a factor. In general, expectations affect the feelings of individuals about themselves and the world around them ^8^. Unrealistic expectations can lead to emotional costs, like lower self-esteem, regret, stress, a feeling of disappointment or a mental imbalance in personal ‘investments’ and ‘returns’ ^9– 11^. These feelings can cause emotional and cognitive impairment or exhaustion, which are the key characteristics of burnout. It is therefore not surprising that several studies in different populations have indicated that there is a correlation between expectations and burnout. *Browning et al.* found that unmet expectations of control were a predictor of burnout in nurses in the USA and *Kim et al.* described a correlation between unmet expectations and burnout in social workers ^12, 13^. Finally, a longitudinal study of *Hultell et al.* suggested a causal relationship between unmet expectations and burnout in teachers when entering employment ^14^.

However, to our knowledge, no previous study has investigated the relationship between unmet expectations and burnout symptoms for medical students. Therefore, this study aims to investigate the relationship between expectations of medical students and burnout symptoms. In order to do so, we explored expectations of medical students regarding their clerkships, as for most students this is their first encounter with the day-to-day aspects of the profession they have been studying for. Subsequently, this study examined whether these expectations were met in reality and if unmet expectations were related to burnout symptoms.

## Methods

### Context

This study was conducted between October 2 and December 22 2023 at the Radboud University Medical Center Nijmegen, the Netherlands. In the Netherlands, medicine is an academic study with a duration of six years. The first three years (bachelor) is mostly theoretically oriented. During the last three years (master) students follow different clerkships at the main medical specialties. These clerkships take place at university hospitals, affiliated hospitals and primary care facilities and have a duration of two to twelve weeks. Between different clerkships students follow educational courses that prepare them for the upcoming clerkship. An overview of our curriculum with the educational courses is published elsewhere ^15^.

### Study design

We used an exploratory mixed methods design, that consisted of two sequential data collection methods. During the first part of the study individual semi-structured interviews were conducted with six students to collect a variety of (unmet) expectations regarding the clerkships that exist among our students. In the second part of this study, these expectations were used to design an anonymous questionnaire. This questionnaire examined the presence of different expectations and the presence of burnout symptoms, so correlations between these measures could be provided.

### Part 1: Exploratory interviews

***Participants***

We used the educational courses between the different clerkships to approach potential respondents. As we expected that students in the later stages of the clerkships would struggle to recall a diversity of expectations, we only approached students that were in their first year of the clerkships. Within this first year we selected three educational courses to approach the students:

- The educational course at the start of the clerkships, as these students did not have any experience with clerkships, we expected them to provide us with the most ‘pure’ expectations.
- The educational course after the first clerkship, as we expected that these students would be able to make the most clear comparison between expectations and reality.
- The educational course at the end of the first year, as we expected that sometimes time is needed to discover that experiences are not met in reality.

The first researcher (AR) visited educational meetings of these three courses to invite students to participate in an in-depth interview about their expectations (and experiences) of the clerkships. This resulted in the participation of six students, two from each educational course.

***Interview process***

We developed a semi-structured interview guide based on our research aim. This guide contained three questions:

1. What were your expectations of the clerkships before you started?
2. To what extent did your expectations of the different clerkships meet reality?
3. If an expectation differed from reality, how did this affect you?

All interviews were performed by one member of the research team (AR) in Dutch. The first of the six interviews was observed by another member of the research team (RG), in order to discuss whether the interview guide provided the necessary information. All interviews were held in a private office, lasted approximately 30 minutes and were audio recorded. Participants provided informed consent before start of the interviews. The interviews were transcribed verbatim.

***Data analysis***

The transcripts were analyzed by the research team, using the constant comparative analysis technique to interpret the data qualitatively whilst also systematically looking at potential relationships between codes ^16^: In order to do so, coding followed a three-part process ^16^: 1) Open coding of all of the interviews by one of the researchers (AR), used to develop an initial template of codes. 2) Independently open coding by two researchers (AR, RG) followed by identifying focal topics. 3) Interpretations, disagreements and doubts about focal topics were discussed among the research team (AR, RG, MP). Through constant further reducing and recoding during multiple sessions with the research team, final topics were identified. Verbatim transcripts of the interviews and the subsequent coding documents are available in our online data collection at https://doi.org/10.34973/dthp-0748 version 2

### Part 2: Questionnaire

***Questionnaire development***

We developed an anonymous questionnaire to fulfil our research aim (see Table 2). This questionnaire consisted of three parts, on which we will elaborate below: demographics, Expectation Reality Mismatch (ERM) and burnout symptoms.

**Demographics.** Students were asked to provide their age and gender.

**ERM.** The topics that were identified during analysis of the interview transcripts provided the outline for this part of the questionnaire. For each topic students were asked if they had any expectations concerning this topic prior to their clerkships. If they had expectations they were asked to indicate how this expectation played out in reality on a 6-item Likert scale (e.g. way worse than expected, worse than expected, as expected, better than expected, way better than expected or did not have any expectations).

In addition to these topics, students were asked to reply to the following statement ‘my expectations before start of the clerkships fully correspond with reality’ by use of a 5-item scale (totally agree, agree, neutral, disagree, totally disagree). The answers to this question were used as our ‘general ERM score’.

Finally, two open-ended questions were formulated. Students were first asked to describe their expectations of the clerkships and, secondly, to describe their actual experiences.

Before implementation, independent researchers and medical students were asked to give feedback on the comprehensibility and the structure of the questions.

**Burnout symptoms.** To measure burnout symptoms, the short Burnout Assessment Tool (sBAT) was used ^17^. The sBAT is a 12-item questionnaire that is a short version of the Burnout Assessment Tool (BAT). It provides a general burnout score and assesses the four core dimensions of burnout (mental distance, cognitive impairment, exhaustion and emotional impairment). We used the sBAT instead of the longer BAT, because it has the same reliability and validity and we did not want to burden our respondents unnecessarily. At this moment, the Maslach Burnout Inventory (MBI) and not (yet) the (s)BAT is considered the “gold standard” for measuring burnout ^18, 19^. However, the sBAT has significant advantages over the MBI: it provides a total-score reflecting the risk of burnout and it includes a cognitive domain, which is an important feature of burnout ^17^.

### Participants and procedure

This questionnaire was carried out in a sample of 155 medical students undertaking their clerkships. Participants were approached during the educational courses in between their clerkships. After classes, students were asked to voluntarily fill out a paper version of the questionnaire.

### Data analysis

All statistical calculations were performed using SPSS Version 27 software ( Downloading IBM SPSS Statistics 27). Basic statistical analysis was performed to calculate the descriptive statistics of expectations and burnout. In order to explore an association between ERM and burnout symptoms the Spearman’s correlation coefficient was calculated for the ‘general ERM score’ and the total sBAT score.

The results from the open-ended questions were used to identify narratives that could extend and/or clarify the topics that were identified during the first part of the study. In order to do so, the open-ended questions were analyzed in the same way as the individual interviews.

### Ethics and consent

The Central Medical Ethical board region Arnhem-Nijmegen ethical review board declared that the study participants are not subjected to acts that are subject to the Medical Research Involving Human Subjects Act (WMO). On this basis, the Central Medical Ethical board region Arnhem-Nijmegen (CMO) declared that the research does not fall under the remits of the WMO (Dossier number: 2020-6518, approval date may 19, 2020). Subsequently the research protocol was approved by the Institutional Research Board of the faculty of Medicine Nijmegen (COMOS-4707672, approval date September 19, 2023). All participating gave written informed consent that their provided demographic data and input during the interviews and questionnaires may be used for publication. The research was conducted in accordance with the Netherlands Code of Conduct for Research Integrity ( Netherlands Code of Conduct for Research Integrity | NWO) and carried out according to the principles of the declaration of Helsinki.

### Data availability

All underlying data of this study are available at: https://doi.org/10.34973/dthp-0748 version 2

## Results

### Part 1: Exploratory interviews

Through analysis of the interview transcripts, ten topics concerning the expectations of the clerkships were identified. These topics, their underlying codes and a representative quote for each respective topic can be found in Table 1.

**Table 1.** The topics and codes that were established during analysis of the interviews with an illustrative quote.

Topics	Codes	Quotes
Amount of time dedicated to clerkships	• Travel time • Self-study • Time spent on clerkship itself	*“I expect that it will take loads of time, especially when I have to * *commute to a hospital faraway”*
Experienced time for relaxation	• Sport • Social contacts • Side job	*“I expect that I will have time for sport, but that a side job will be too * *much for me”*
Supervision during clerkships	• Supervision on individual work • Involving student in daily practice • Possibility to ask questions • Multiple and fast changing of supervision	*“The doctor did not look at me, walked out of the room without * *saying where she went, answered my questions very briefly, and did * *not involve me at all… This is not what I expected”*
Quality of feedback	• Content quality/value of feedback • Opportunities to ask feedback • Quantity of feedback	*“A surgeon once gave me the feedback: ‘don’t get in the way’. I did * *not expect this”*
Amount of time spent on different tasks	• Patient contact • Administration • Observing doctor • Education	*“I expected more one-on-one patient contact and did not realize that * *a big part of the afternoon would consist of administration”*
Amount of initiative needed	• Dealing with a low patient flow • Assertiveness	*“I experienced that the cliché ‘you have to create your own clerkship’ * *was true”*
Emotional impact of experiences	• Frequently switching clerkships • Psychological impact of specific patient cases • Amount of impressions	*“I did not expect this could happen to me: the only thing I could do * *for the entire weekend was think about that patient”.*
Culture on clerkship location	• Hierarchy • Overall ambiance • Accessibility of colleagues	*“The culture in the hospital was better than I expected: there was * *almost no hierarchy”*
General educational outcomes	• Practical skills • Professional conduct • Medical knowledge	*“I expect that I am going to have a very steep learning curve”*
Pressure to perform	• Perfectionism • Comparison with other students	*“I never had problems during my study, but because I was now doing * *my clerkship together with a very smart student, I felt a very high * *pressure to perform: I also wanted to give the correct answers to the doctor”*

Multiple topics are related to each other (e.g. amount of time dedicated to clerkships and experienced time for relation could be seen as interdependent). Consequently, there was a possibility to further reduce the topics (e.g. time investment). However, we considered these concrete topics more suitable to include in the questionnaire than abstract, overarching themes, as we expected the students to have a better sense of the topics.

### Part 2: Questionnaire

All of the 155 students that were asked to participate filled in the questionnaire. The participants’ age range was 21 to 29 (M=23.89, SD=1.55) and 71.0% of the participants identified as women, 27.7% as men and 1.3% as other.

Table 2 shows the extent of (mis)match between expectations and reality that the participants reported for each of the ten topics that were identified during the first part of the study. All students indicated that they had expectations regarding the ten topics of the questionnaire prior to their clerkships. The experienced time for relaxation, level of supervision, quality of feedback, amount of time spent on different tasks and pressure to perform were experienced less or worse than expected. On the other hand, the amount of initiative needed and the culture on clerkship location were experienced more or better than expected.

**Table 2.** The experienced (mis)match between expectations and reality per topic (N= 155).

Topic	Way less/ worse than expected n (%)	Less/worse than expected n (%)	As expected n (%)	More/better than expected n (%)	Way more/ better than expected n (%)	No expectations n (%)
**Amount of time ** **edicated to ** **clerkships**	0 (0%)	24 (15.5%)	77 (49.7%)	44 (28.4%)	2 (1.3%)	8 (5.2%)
**Experienced time ** **for relaxation**	9 (5.8%)	63 (40.6%)	40 (25.8%)	33 (21.3%)	1 (0.6%)	9 (5.8%)
**Supervision ** **during clerkships**	9 (5.8%)	42 (27.1%)	40 (25.8%)	23 (14.8%)	0 (0%)	41 (26.5%)
**Quality of ** **feedback**	16 (10.3%)	61 (39.4%)	24 (15.5%)	7 (4.5%)	0 (0%)	47 (30.3%)
**Amount of time ** **spent on different ** **tasks**	3 (1.9%)	37 (23.9%)	40 (25.8%)	17 (11.0%)	1 (0.6%)	57 (36.8%)
**Amount of ** **initiative needed**	0 (0%)	2 (1.3%)	31 (20.0%)	67 (43.2%)	23 (14.8%)	32 (20.6%)
**Emotional impact ** **of experiences**	6 (3.9%)	40 (25.8%)	49 (31.6%)	22 (14.2%)	3 (1.9%)	35 (22.6%)
**Culture on ** **clerkship location**	4 (2.6%)	15 (9.7%)	50 (32.3%)	50 (32.3%)	3 (1.9%)	33 (21.3%)
**General ** **educational ** **outcomes**	1 (0.6%)	33 (21.3%)	68 (43.9%)	37 (23.9%)	3 (1.9%)	13 (8.4%)
**Pressure to ** **perform**	4 (2.6%)	19 (12.3%)	45 (29.0%)	57 (36.8%)	6 (3.9%)	24 (15.5%)

Table 3 shows the responses of participants in concern to the general ERM score, which was based on the statement ‘my expectations before start of the clerkships fully correspond with reality’. All participants did have expectations about the clerkships before they started and 65.1% of the students (totally) disagreed with the statement. Furthermore, the table provides the sBAT-scores of the participants, which are divided into four categories (low, average, high and very high). These categories are based on statistical norms of Dutch employees. The table shows that 21.9% of the students had a high to very high sBAT-score, meaning that the total sBAT score was at least 2.80 ^17^. The average total sBAT-score was 2.36 (SD=0.62).

**Table 3.** The general ERM score * and sBAT-score of the participants (N=155).

Questionnaire item	n (%)
**The general ERM** ** - Totally agree** ** - Agree** ** - Neutral** ** - Disagree** ** - Totally disagree** **Did not have any expectations**	0 (0%) 31 (20%) 23 (14.8%) 94 (60.6%) 7 (4.5%) 0 (0%)
**sBAT-score (statistical norms of Dutch ** **employees)** ** - Low (1.00-1.50)** ** - Average (1.51-2.79)** ** - High (2.80-3.66)** ** - Very high (3.67-5.00)**	14 (9.6%) 100 (68.5%) 26 (17.8%) 6 (4.1%)

*The general ERM is based on the following statement: ‘my expectations before start of the clerkships fully correspond with reality’.

The Spearman’s correlation coefficient of the general ERM and the total sBAT-score was 0.264 (p<0.001), meaning that students who reported a bigger mismatch between expectations and reality had a higher total sBAT-score.

In the open-ended questions of the questionnaire students reported that their experiences differed a lot per clerkship and per clerkship location. This may explain the high degree of variation that is visible in Table 2. In addition, the answers to these questions helped us to elaborate on five of the ten topics that were identified during the first part of the study: supervision during clerkships, quality of feedback, amount of time spent on different tasks, amount of initiative needed and culture on clerkships location.

Participants described an evident mismatch between expectations and reality of the supervision at the clerkships. They expected direct and personal supervision on a regular basis in a safe learning environment. Moreover, participants expected that they would be part of the team. In reality, participants often missed personal, direct or scheduled supervision and did not experience being part of the team:

*“On a regular basis, I had the feeling that I could disappear without anyone noticing.”* (Participant 53)

Participants expected to receive a lot of personal, good quality feedback, based on specific situations. In practice, participants experienced that the quality of feedback was inconsistent, due to large differences between supervisors. Not rarely, participants were confronted with impersonal and unspecific feedback:

*“I often fill in my feedback form about a situation myself, the supervisor approves it afterwards without having been present.”* (Participant 14)

Regarding the amount of time spent on different tasks, participants expected to have a lot of direct patient contact. They anticipated that especially in the beginning of the clerkships a part of this contact would consist of watching the doctor and patient, but they also expected to have some autonomy with regard to their time and tasks. The majority of the participants described that there was less direct patient contact, autonomy and attention for practical skills than expected. However, other participants experienced more autonomy than expected:

“ *There is limited opportunity to learn practical skills such as taking a blood sample or perform an iv-puncture...”.* (Participant 124)

Participants did expect that they would have to initiate some activities, but also that there would be a proper introduction and some structure. In reality, participants experienced that there were little opportunities to participate if they did not ask for this:

*“I needed more initiative than expected. If you don’t say anything, you don’t exist.”* (Participant 97)

In concern to the culture on the clerkship location, participants expected a strict hierarchy and unkind doctors. During their clerkships, however, participants noticed that hierarchy was less present than expected and that most doctors were friendly. 

*“The doctors can be quite friendly and less judgmental.”* (Participant 24)

## Discussion

This study aimed to explore the relationship between expectations concerning the clerkships and burnout symptoms in medical students. Therefore, we developed a questionnaire that measured the degree of mismatch between expectations and reality regarding the clerkships of medical students and the extent to which these students experienced burnout symptoms. The results of this questionnaire showed that ERM was positively correlated with burnout symptoms in medical students. In other words, those students that experienced a bigger mismatch between expectations and reality also experienced more burnout symptoms. 

There are several explanations of the correlation between ERM and burnout symptoms. Firstly, an explanation could be that a mismatch between expectations and experiences causes burnout symptoms. Unrealistic optimism can lead to negative emotional consequences, like lower self-esteem and wellbeing, when the expectations are not met ^9^. Consequently, these feelings can possibly lead to burnout symptoms. Another explanation of the correlation between ERM and burnout symptoms could be that students experience a bigger ERM as a result of burnout symptoms (reverse causality). Since burnout syndrome includes emotional and cognitive disruption, students with a high degree of burnout symptoms may be more prone to experience a mismatch between their expectations and reality in retrospect, while in fact their initial expectations did correspond with reality.

Finally, ERM and burnout symptoms could both be the result of a third variable. Students stated that the level of supervision, the quality of feedback and the amount of time spent on different tasks did not meet their expectations. Interestingly, good feedback, people-orientated supervision and a diverse work-environment are known to be protective factors for the development of burnout syndrome ^10, 20, 21^. The absence of these factors could independently result in an ERM and burnout symptoms.

In this study 21.9% of the participants scored high to very high on the sBAT. The statistical norm of a high to very high burnout level on the sBAT was based on the 75 ^th^ percentile of a representative sample of Dutch employees, meaning that 25% of this population scored high to very high ^17^. Therefore, our findings seem to contradict earlier studies that indicate that the prevalence of burnout (symptoms) is significantly higher for medical students compared to the general population ^3– 5^.

However, it is important to bear in mind that the prevalence of burnout symptoms among medical students is very heterogeneous, due to the use of different measurement scales, varying cutoff scores and the cultural and educational differences per country or university ^3, 22, 23^. Moreover, a high degree of burnout symptoms is not equivalent to the presence of burnout syndrome ^24– 26^.

While keeping these concerns in mind, the correlation between ERM and burnout symptoms is still of relevance to medical educators, as it indicates that an experienced mismatch between expectations and reality can color our current experiences and emotions negatively. Consequently, expectation management prior and/or during the clerkships could be an interesting avenue to reduce negative experiences and emotions during the clerkships.

### Strengths and limitations

An important strength of this study is the response rate of 100%, as this provided us with a representative sample of the medical students that follow their clerkships. Furthermore, the mixed methods approach combined in-depth information regarding the type of expectations that medical students have with comprehensive information concerning the presence of these expectations and burnout symptoms.

Additionally, also some limitations of this study deserve attention. Firstly, causality between ERM and burnout symptoms could not be identified as a result of the cross-sectional design. Therefore, future prospective longitudinal research is needed to confirm causality. Furthermore, the closed-ended question on which the ERM-score was based only focused on a mismatch between expectations and reality, but did not tell us if reality was better or worse than expected. We specifically chose for this method, because some experiences cannot easily be classified as better or worse than expected. However, the difference between experiences that are better or worse than expected can be of interest in future research.

## Conclusions

In conclusion, this exploratory study showed that a mismatch between expectations and experiences of medical students regarding the clerkships is positively correlated with burnout symptoms. It is important that future research further establishes this relationship, as expectation management might be an interesting avenue to reduce negative experiences and emotions during the clerkships.

## List of abbreviations

ERM: Expectation Reality Mismatch

sBAT: short Burnout Assessment Tool

## Ethics and consent

The Central Medical Ethical board region Arnhem-Nijmegen ethical review board declared that the study participants of this study are not subjected to acts that are subject to the Medical Research Involving Human Subjects Act (WMO). On this basis, the Central Medical Ethical board region Arnhem-Nijmegen the Netherlands (CMO) declares that the research does not fall under the remits of the WMO (Dossier number: CMO-020-6518). Subsequently the research protocol was approved by the Institutional Research Board of the faculty of Medicine Nijmegen (Dossier number COMOS-4707672). All participating students gave written informed consent. All data were anonymized, no identifying information is present. The research was conducted in accordance with the Netherlands Code of Conduct for Research Integrity ( Netherlands Code of Conduct for Research Integrity | NWO) andcarried out according to the principles of the declaration of Helsinki.

## Consent for publication

Not applicable.

## Data Availability

Radboud University- A mixed methods study on the association between burnout symptoms and expectations regarding clerkships in Dutch medical students. https://doi.org/10.34973/dthp-0748 ^27^. The project contains the following underlying data: Data file 1. ABOUT.txt Data file 2. Explanatory file data new1.pdf Data file 3. LICENSE.txt Data file 4. MANIFEST.txt Data file 5. Protocol interview Dutch.pdf Data file 6. UMC Checklist version 1.2.docx Data file 7. Informed consent formulier Eng NL.pdf Data file 8 questionnaire Dutch.pdf Data file 9 Codering inetviews NL.pdf Data file 10 Coding interviews ENG.pdf Data file 11 Verslag interviews student 1–6 NL anoniem Data file 12 Dataset questionnaire.xlsx Data file 13 Interview protocol english.pdf Data file 14 Interview students english anonymous.pdf Radboud University- A mixed methods study on the association between burnout symptoms and expectations regarding clerkships in Dutch medical students. https://doi.org/10.34973/dthp-0748 ^27^. All data are available as underlying data, no extra extended data

## Explanatory information about the data availability

Availability at: A mixed methods study on the association between burnout symptoms and expectations regarding clerkships in Dutch medical students (ru.nl)

https://doi.org/10.34973/dthp-0748 version 2

Title corresponding article: : A mixed methods study on the association between burnout symptoms and expectations regarding clerkships in Dutch medical students Authors: Rubens, (A)Bram; Gulden, Rozemarijn van der; Pol, Marjolein van de

- Background: Over half of the Dutch students experience burnout symptoms. In order to design effective interventions, it is important to know more about cognitions and behaviors that relate to burnout. An example of such cognitions might be inaccurate expectations of medical students regarding their study, as studies in other populations already indicated that unmet expectations can relate to burnout symptoms. Therefore, the aim of this study is to evaluate the relationship between an expectation-reality mismatch of medical students and burnout symptoms. Methods: We performed an exploratory mixed methods study consisting of two sequential data collection methods. Firstly, individual semi-structured interviews were performed with six students to explore expectations. Secondly, we designed an anonymous questionnaire to explore presence of expectation mismatches and the relationship with burnout symptoms. Burnout was assessed using the short Burnout Assessment Tool (sBAT). The data in this folder consist of Data file 1. ABOUT.txt, Data file 2. Explanatory file data new1.pdf, Data file 3. LICENSE.txt, Data file 4. MANIFEST.txt, Data file 5. Protocol interview Dutch.pdf, Data file 6. UMC Checklist version 1.2.docx, Data file 7. Informed consent formulier Eng NL.pdf, Data file 8 questionnaire Dutch.pdf, Data file 9 Codering inetviews NL.pdf, Data file 10 Coding interviews ENG.pdf, Data file 11 Verslag interviews student 1-6 NL anoniem, Data file 12 Dataset questionnaire.xlsx, Data file 13 Interview protocol english.pdf, Data file 14 Interview students english anonymous.pdf
