# Supplementary material for: A mixed methods study on the association between burnout symptoms and expectations regarding clerkships in Dutch medical students
Source: MedEdPublish (2016). 2025 Jul 18;15:34. [Version 1] doi: 10.12688/mep.20633.1 (PMC12640506; doi:10.12688/mep.20633.1)
Supplement: Peer review history

# Reviewer report: Reviewer response for version 1

**DOI:** 10.21956/mep.22088.r44325

**Published:** 2025-11-22

**Authors:** Rhoda Meyer, Stella Goeschl

**Affiliations:** 1 Stellenbosch University, Cape Town, Cape Town, South Africa; 2 Imperial College London, London, London, UK

Dear authors,

 Thank you for your submission. The study is timely, given the high level of burnout amongst medical students, and has the potential to contribute to the discourse. The problem of medical students’ unmet expectations regarding their studies and the link to burnout is highlighted, showing the necessity of this study in the context of medical education.  The mixed method design, including semi-structured interviews and a questionnaire, is relevant to exploring students’ expectations. The findings confirm that there is a strong link between expectations and burnout.

 ​​​​​​There are opportunities to improve this submission. Please find reviewer comments below.

 Abstract:

- “Over half of the Dutch students” – please specify whether medical students, specify whether university or school students
- While the issue of unmet expectations is highlighted, it is important to say why this study is necessary in your context. Perhaps the context is unique to those where previous studies were done? If this is the case, then the uniqueness of your context needs to be made more explicit. Adding more on context allows the reader to decide whether the study is of relevance to them.
- Methods should briefly mention the target population for the questionnaire and how the analysis was planned or conducted.
- Please also indicate the type of mixed methods study. Was it a sequential exploratory or a sequential explanatory study? And did the qualitative interviews inform the questionnaire? If not, where did the integration take place?
- For the conclusion section, it is important to add one or two recommendations to show how the study contributes to the field.

Background:

- Good explanation of rationale and relevant literature
- The opening sentence provides a broad view of the problem, but it is important to say whether this is in the context of higher education, or medical education specifically.
- While it is true that cognitions and behaviours can relate to burnout, you also need to acknowledge other factors first, and then indicate that the focus of this study is on cognition and behaviours only.
- Since this study is on burnout, the different ways in which it is conceptualised needs to be explored. We would also recommend that you delve deeper into the consequences of burnout for the student, peers, team, and patient.
- It is good that you have attempted to indicate the gap (which is no previous study), however, this is not sufficient to conduct a study. A stronger justification is needed, for example, why is it important to explore this in your context? What will be the effects if this study is not conducted? Will the failure rate be higher? Or will more students experience mental health issues?
- We would also recommend that you add a theory to explain how unmet expectations link to burnout, to strengthen the theoretical foundation of your study, for example, self-determination theory.
- We noticed that there was no research question, aims and objectives for this study. These need to be included to focus your study and establish its boundaries.

Methods:

- A description of the context is provided, but more is needed in terms of the factors that could influence how students respond to stress or report symptoms of burnout.
- You have explained that a researcher visited the educational meetings to recruit students. Please indicate the relationship of the researcher to the students. Were there any possibilities of students feeling coerced into participating, and how was this mitigated to protect participants?
- Please include what informed the interview questions. Did you use the literature to inform this or was this based on another interview instrument?
- Interview process: does not clarify how the questions were adapted to those students who had not yet started clerkships (from the educational course at the start of the clerkships)
- Potential selection bias for questionnaire, potential bias through self-reporting of burnout symptoms; does not clarify during which of the educational courses students were approached (also the one at the beginning and end of the first year like interviewees?); would be useful to also clarify clerkship years of survey respondents and provide a number for the typical year cohort to set the 155 into context

Results:

- Table 1: analysis blends both expectations and realities into a single analysis; it may lend itself to better interpretability in the context of the research question to list these separately
- “All students indicated that they had expectations regarding the ten topics of the questionnaire prior to their clerkships”; however, Table 2 lists several students per category for “no expectations”
- Table 2: blending of the categories, such as in “more / better than expected” or listing in this way, is contradictory / confusing: i.e., “amount of initiative needed”, where “more” would mean more work, which is not necessarily “better; same with the less/worse categories; please clarify or adapt wording
- Only limited integration of the burnout component into the results. Since the purpose of the study was to explore the link between the two elements, this integration is critical. Please revise the results section to show how burnout is linked to unmet expectations.
- Table 3: formatting not aligned for sBAT
- sBAT scores could be explained further beyond Dutch employees’ reference – does “low” and “average” mean burnout symptoms are still present but below pathological level, or not present at all?

Discussion:

- Good integration of results and mention of potential confounders. However, in certain areas, strong claims are made without adequate justification. For example, it is mentioned that ‘those students who experienced a bigger mismatch between expectations and reality also experienced more burnout symptoms’, but the statements following this are not supported by your findings.
- It is mentioned that burnout symptoms among medical students is heterogeneous and is influenced by the culture and context. It is thus important to describe the context of your study so that readers can understand the impact of your context on your findings.
- Response rate of 100% - could be mentioned earlier
- There are no clear recommendations offered for other medical schools. The value of this study is therefore questionable as it Is not clear how it adds to the discourse in the field.

References:

 Largely current literature, but few from recent years

Have any limitations of the research been acknowledged?

Yes

Is the work clearly and accurately presented and does it cite the current literature?

Partly

If applicable, is the statistical analysis and its interpretation appropriate?

I cannot comment. A qualified statistician is required.

Are all the source data underlying the results available to ensure full reproducibility?

Yes

Is the study design appropriate and is the work technically sound?

Partly

Are the conclusions drawn adequately supported by the results?

Yes

Are sufficient details of methods and analysis provided to allow replication by others?

Partly

Reviewer Expertise:

Health professions education, qualitative research

We confirm that we have read this submission and believe that we have an appropriate level of expertise to confirm that it is of an acceptable scientific standard, however we have significant reservations, as outlined above.

# Reviewer report: Reviewer response for version 1

**DOI:** 10.21956/mep.22088.r44322

**Published:** 2025-11-22

**Authors:** I Made Moh. Yanuar Saifudin

**Affiliations:** 1 Universitas Tanjungpura, Pontianak, Indonesia

This mixed methods study addresses an important and timely topic: the relationship between expectation-reality mismatch (ERM) and burnout symptoms in Dutch medical students during clerkships. The study is methodologically sound with an innovative approach combining qualitative interviews and quantitative survey data. However, several areas require clarification and expansion to strengthen the manuscript's contribution and clinical applicability.

 Abstract:

 - Results: The correlation coefficient (r=0.264, p<0.001) should be included in the abstract for transparency.

 - Conclusions: The phrase "future research further establishes this relationship" is somewhat vague. Consider strengthening: "Future longitudinal research should establish causality, as expectation management interventions may reduce burnout symptoms during clerkships"

 Introduction:

 The introduction would benefit from a brief theoretical explanation of why clerkships specifically might create expectation mismatches. Consider adding 1-2 sentences: "Clerkships represent students' first sustained exposure to clinical practice, where abstract medical knowledge meets complex realities of patient care, healthcare systems, and professional culture. This transition may be particularly vulnerable to expectation mismatches."

 Methods

 - Line 59: "Between different clerkships students follow educational courses" - briefly clarify the purpose/content of these courses (e.g., "preparatory educational courses covering clinical skills and specialty-specific knowledge")

 - Part 1: Exploratory Interviews. Table with basic demographics of the 6 interviewees would be helpful (gender, which clerkship completed if applicable, clerkship location type). This helps readers assess diversity of perspectives captured

 - Part 2: Questionnaire. Who were the "independent researchers and medical students" who reviewed it? How many? What specific feedback was incorporated?

 Results:

 In open-ended question results, it’s good elaboration on 5 of the 10 topics. However, why these 5 topics? Were they; Most frequently mentioned? Most strongly correlated with burnout? Most novel/unexpected?

 Discussion:

 - The discussion addresses main findings but lacks depth in interpretation and misses opportunities to explore implications.

 - You mention that absence of good supervision, feedback, and diverse work are protective factors. These are not just mediators - they may be the actual problem. Perhaps ERM is simply a marker for poor clerkship quality. You may strengthen the discussion by mentioning: "Our findings suggest that ERM may serve as a proxy measure for clerkship quality. Students expected good supervision and feedback because these are fundamental educational principles. When clerkships fail to provide these, students experience both unmet expectations and the direct consequences of poor learning environments—both of which contribute to burnout."

 - This is only 6 lines discussing implications, despite being the practical contribution of your work.

 Overall:

 This study addresses an important and novel question with an appropriate mixed methods design and achieves strong recruitment. The correlation between ERM and burnout is a valuable finding with potential implications for medical education. However, major revision is needed, so this manuscript could make an important contribution to understanding medical student burnout and informing educational interventions.

Have any limitations of the research been acknowledged?

Partly

Is the work clearly and accurately presented and does it cite the current literature?

Yes

If applicable, is the statistical analysis and its interpretation appropriate?

Yes

Are all the source data underlying the results available to ensure full reproducibility?

Partly

Is the study design appropriate and is the work technically sound?

Yes

Are the conclusions drawn adequately supported by the results?

Partly

Are sufficient details of methods and analysis provided to allow replication by others?

Yes

Reviewer Expertise:

Nursing

I confirm that I have read this submission and believe that I have an appropriate level of expertise to confirm that it is of an acceptable scientific standard, however I have significant reservations, as outlined above.

# Reviewer report: Reviewer response for version 1

**DOI:** 10.21956/mep.22088.r42829

**Published:** 2025-09-02

**Authors:** Ferry Dwi Kurniawan

**Affiliations:** 1 Syiah Kuala University, Banda Aceh, Indonesia

Dear Authors

 I appreciate for your hard work. However , there are some parts that can be improved in the manuscript.

 DISCUSSION

 "this exploratory study showed that a mismatch between expectations and experiences of medical students regarding the clerkships is positively correlated with burnout symptoms"

 Please highlight the cause factors extracted from interviewed. And please put it in the conclusion  

 LIMITATION

 "causality between ERM and burnout symptoms could not be identified as a result of the cross-sectional design"

 Yes, indeed. Please identify the confounding factors that may exist and please add in the manuscripts.

Have any limitations of the research been acknowledged?

Partly

Is the work clearly and accurately presented and does it cite the current literature?

Yes

If applicable, is the statistical analysis and its interpretation appropriate?

Yes

Are all the source data underlying the results available to ensure full reproducibility?

Yes

Is the study design appropriate and is the work technically sound?

Yes

Are the conclusions drawn adequately supported by the results?

Partly

Are sufficient details of methods and analysis provided to allow replication by others?

Yes

Reviewer Expertise:

Please add the minor revisions in the manuscript.

I confirm that I have read this submission and believe that I have an appropriate level of expertise to confirm that it is of an acceptable scientific standard.
